# Tracing the Photoaddition of Pharmaceutical Psoralens to DNA

**DOI:** 10.3390/molecules25225242

**Published:** 2020-11-10

**Authors:** Janina Diekmann, Isabell Theves, Kristoffer A. Thom, Peter Gilch

**Affiliations:** Institut für Physikalische Chemie, Heinrich-Heine-Universität Düsseldorf, Universitätsstr. 1, 40225 Düsseldorf, Germany; janina.diekmann@hhu.de (J.D.); isabell.theves@hhu.de (I.T.); kristoffer.thom@hhu.de (K.A.T.)

**Keywords:** psoralen, 8-MOP, TMP, 5-MOP, DNA damage, PUVA, photochemistry, UV/Vis spectroscopy, IR spectroscopy, time-resolved spectroscopy

## Abstract

The psoralens 8-methoxypsoralen (8-MOP), 4,5′,8-trimethylpsoralen (TMP) and 5-methoxypsoralen (5-MOP) find clinical application in PUVA (psoralen + UVA) therapy. PUVA treats skin diseases like psoriasis and atopic eczema. Psoralens target the DNA of cells. Upon photo-excitation psoralens bind to the DNA base thymine. This photo-binding was studied using steady-state UV/Vis and IR spectroscopy as well as nanosecond transient UV/Vis absorption. The experiments show that the photo-addition of 8-MOP and TMP involve the psoralen triplet state and a biradical intermediate. 5-MOP forms a structurally different photo-product. Its formation could not be traced by the present spectroscopic technique.

## 1. Introduction

**Scheme 1:**
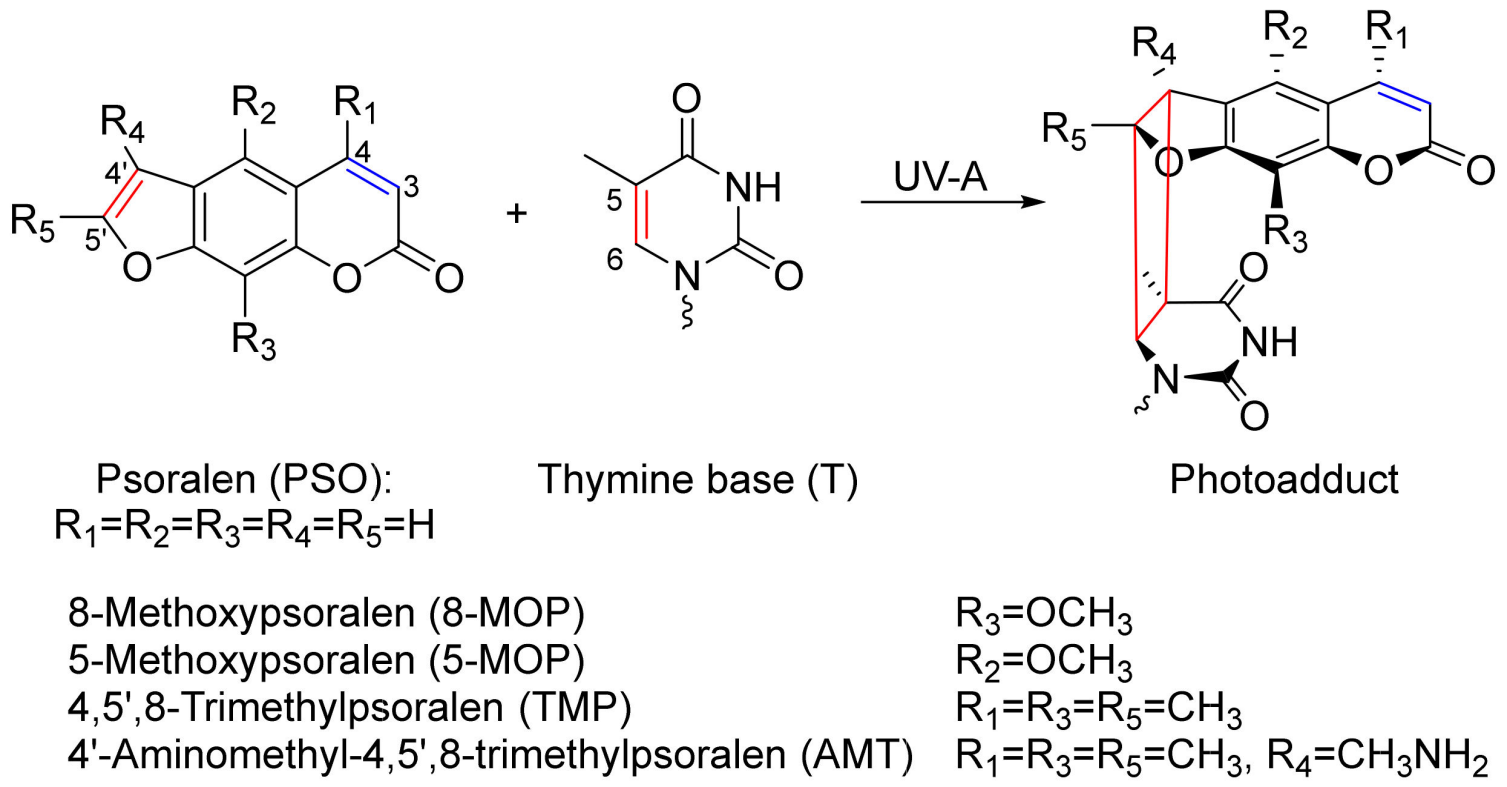
Structures of relevant psoralen derivatives and their photoaddition to DNA. Reaction via the 4′-5′-double-bond results in the furan monoadduct. The DNA is represented by the thymine base.

The light-dependent PUVA (psoralen + UVA) therapy is a well-established symptomatic treatment of skin diseases like psoriasis [1,2], atopic eczema [3], vitiligo [4,5] and cutaneous T-cell lymphoma [6,7]. In the treatment, patients are administered psoralen derivatives and the affected skin regions are exposed to UVA radiation [8]. Concerning the molecular mechanism of the therapy, it was shown that the uptake of psoralens in the cellular nuclei is the first step [9]. While the main targets of the psoralen are the nucleic acids, minor interactions with other biomolecules like lipids and proteins in other parts of the cell can take place [10]. Concerning DNA as a target, there is consensus that psoralens intercalate into DNA, that is, they insert themselves between the base pairs. Upon photo-excitation, the intercalated psoralens may bind to the DNA base thymine (see Scheme 1). A cyclobutane ring forms involving the 4′ and 5′ positions of the psoralen (furan side) and the five and six positions of the thymine base [11,12,13]. The formation of a cyclobutane ring involving the three and four positions of the psoralen (pyrone side) was also found. The ratio of the two adducts depends on the substitution pattern [14]. Photo-excitation of the furan side adduct can trigger another photoaddition which results in DNA crosslinking. The damage done to DNA by (mono-) adducts and crosslinks can induce apoptosis of the affected cells, ultimately resulting in the relief of symptoms [15].

The mechanism of this photoaddition was addressed by steady-state [11] and time-resolved spectroscopy [16,17,18] as well as quantum chemistry [19,20,21,22]. In a recent study, we traced the photo-addition of psoralen to DNA in real-time [23]. The study addressed the derivative 4′-aminomethyl-4,5′,8-trimethylpsoralen (AMT) and a DNA double-strand consisting of alternating adenine (A) and thymine (T) bases (AT-DNA in the following). AMT was selected as it features a high water solubility [24], a relatively high intercalation affinity (see below) and a high quantum yield for the photoaddition of 0.12 [25]. For AMT intercalated into DNA bearing guanine (G) and cytosine (C) base pairs, a photo-induced electron transfer (PET) was observed. This PET reduces the propensity for the photoaddition [26]. Therefore, experiments were conducted with AT-DNA. AMT predominantly forms the furan side adduct. With nanosecond UV/Vis and IR spectroscopy, it was shown that the photo-addition of AMT proceeds via a local triplet state. This state features lifetimes in the range 1–10 µs. The spread is presumably due to heterogeneity of the sample. The decaying local triplet state feeds a triplet biradical in which the AMT moiety is connected with a thymine base via a C-C single bond. Formation of the second bond and thereby the final photo-product takes ~50 µs. AMT has favorable properties for a spectroscopic characterization and potentially for clinical use. However, AMT has no approval for clinical applications. Psoralens which received approval are 8-methoxypsoralen (8-MOP), 4,5′,8-trimethylpsoralen (TMP) and 5-methoxypsoralen (5-MOP) [8]. In most countries, the commonly used psoralen is 8-MOP [7]. While in the United States, 8-MOP is the only derivative available for clinical use [6], in some European countries TMP and 5-MOP find application [1,4]. Yet, TMP and 5-MOP are less studied and only rarely administered [8]. 

Here, it will be investigated in how far the mechanistic picture derived from experiments on AMT can be transferred to psoralens (8-MOP, TMP and 5-MOP) used in clinics. While for AMT a combination of time-resolved UV/Vis and time-resolved infrared (IR) spectroscopy was used to resolve the kinetics of the photo-addition, herein we use solely the technique of time-resolved UV/Vis spectroscopy available in our lab. The small quantum yields and/or water solubilities of these derivatives are very challenging for the small signals in time-resolved IR spectroscopy. It will be shown that for a comparison between derivatives the not as demanding and less resource-intensive method of time-resolved UV/Vis spectroscopy can still show distinct similarities and differences between derivatives. To this end, dissociation constants characterizing the intercalation, reaction quantum yields, spectroscopic patterns as well as kinetic parameters of the photo-addition were recorded. Based on the results, some guidelines for the rational improvement of PUVA agents shall be given. 

## 2. Results

The following results show spectroscopic measurements on psoralen derivatives with and without DNA. Synthetic DNA double strands with alternating adenine (A) and thymine (T) bases, AT-DNA in the following, were employed. They were formed by annealing 5′-(TA)_20_-3′ single strands. All three psoralen derivatives have, as opposed to AMT, low water solubility ranging from few micromolar for TMP to a few hundred micromolar for 8-MOP [27]. The apparent solubility increases when the psoralen can intercalate into DNA [28].

### 2.1. Intercalation

Intercalation of psoralens into DNA is a prerequisite for the photoaddition and, thus, has to be characterized. The propensity for intercalation is commonly quantified by the dissociation constant K_D_ [24]:

(1) $$K_{D}=\frac{c_{Pso,free}\cdot c_{DNA,free}}{c_{Pso,int}}.$$

**Figure 1:**
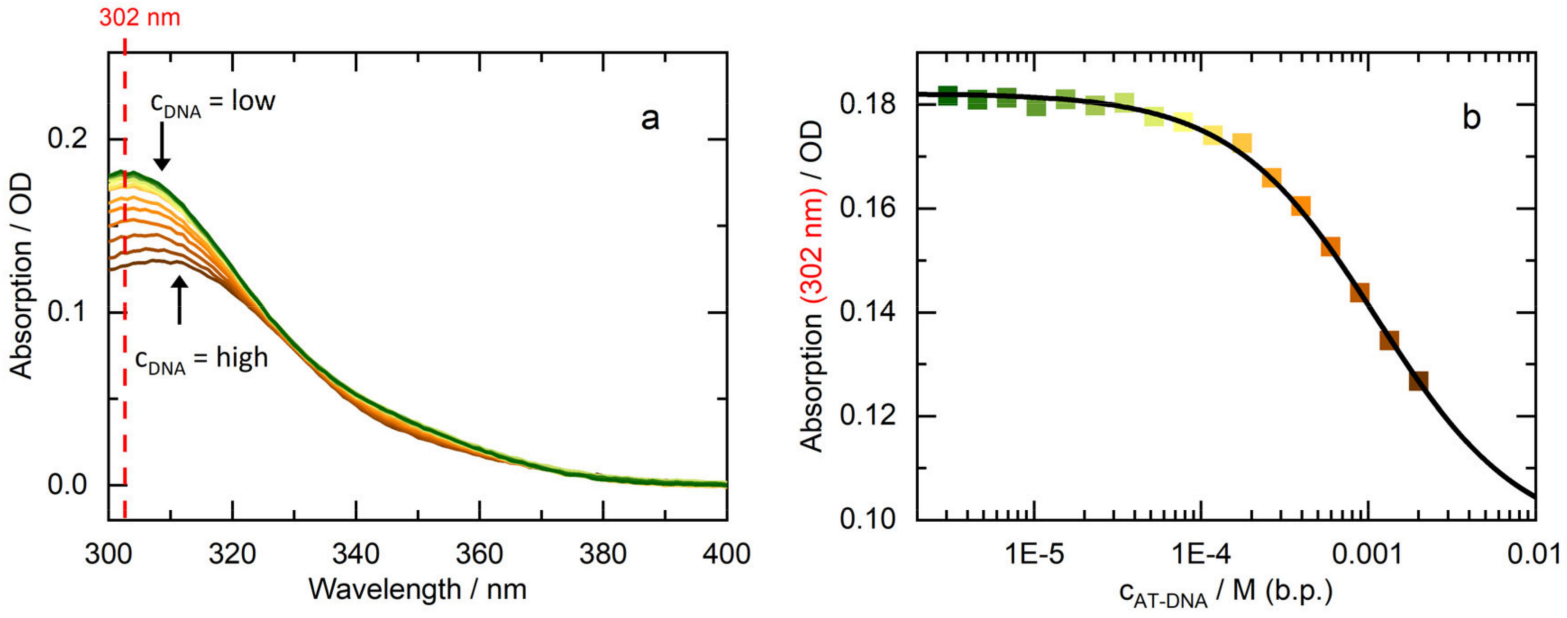
Intercalation behavior of 8-MOP: (**a**) UV/Vis absorption spectra of 8-MOP (15 µM) with increasing concentration of AT-DNA in phosphate-buffered saline (PBS). The contribution of DNA to the absorption was subtracted; (**b**) Absorption at 302 nm versus the concentration of AT-DNA. A fit yields the dissociation constant K_D_ of 1.1 × 10^−3^ M.

$c_{Pso,free}$ is the concentration of free (non-intercalated) psoralen, $c_{Pso,int}$ stands for the concentration of intercalated psoralen and $c_{DNA,free}$ the concentration of DNA base pairs which are not hosting a psoralen. All concentrations refer to equilibrium conditions. A small K_D_ value represents a strong intercalation affinity. The dissociation constant K_D_ can be determined by a titration experiment [25,29] which relies on the hypochromic effect in the UV/Vis absorption of psoralens upon intercalation [30]. In the titration, the total concentration of psoralen $c_{Pso,free}+\;c_{Pso,int}$ was kept constant and the total DNA concentration $c_{DNA}$ gradually reduced (see Reference [25] for details). Results of such a titration for 8-MOP and AT-DNA are summarized in Figure 1. In the respective UV/Vis absorption for wavelengths larger than 300 nm—the spectral region below cannot be covered due to the high DNA absorption—the impact of DNA on the 8-MOP absorption is clearly visible. For high DNA concentration and thereby a large fraction of intercalated 8-MOP the absorption is relatively small. For low DNA concentration and thereby mostly free 8-MOP the absorption is higher. This is in line with the hypochromic effect of intercalation. From the dependence of the absorption at 302 nm on the total DNA concentration the dissociation constant K_D_ can be determined. The procedure relies on Equation (1) as well as Beer’s law and is specified in Reference [25]. The K_D_ value of 8-MOP and AT-DNA derived thereby amounts to 1.1 × 10^−3^ M. For 8-MOP and calf thymus DNA, Isaacs et al. have determined a similar value of 1.3 × 10^−3^ M [27]. Deviation in K_D_ values can be expected for differing DNA sequences and ionic strength of the sample [31]. For AMT and AT-DNA, a somewhat smaller value of 4.4 × 10^−4^ M [25] was reported. With the same procedure (data not shown) a dissociation constant K_D_ of 1.8 × 10^−4^ M for 5-MOP and AT-DNA was determined. A higher intercalation affinity compared to 8-MOP is in line with early reports [32]. The low solubility of TMP in water renders a K_D_ determination by the above procedure difficult. Therefore, only the order of magnitude is estimated for the constant K_D_. For this estimate solid TMP was added to a solution of AT-DNA in amounts exceeding its solubility. Under these conditions the concentration $c_{Pso,free}$ ought to equal the saturation concentration of TMP. The concentration of intercalated TMP $c_{Pso,int}$ was determined photometrically. From these values, a K_D_ value of the order of 10^−4^ M was estimated.

### 2.2. UV/Vis Absorption Signatures of the Photoadditions

**Figure 2:**
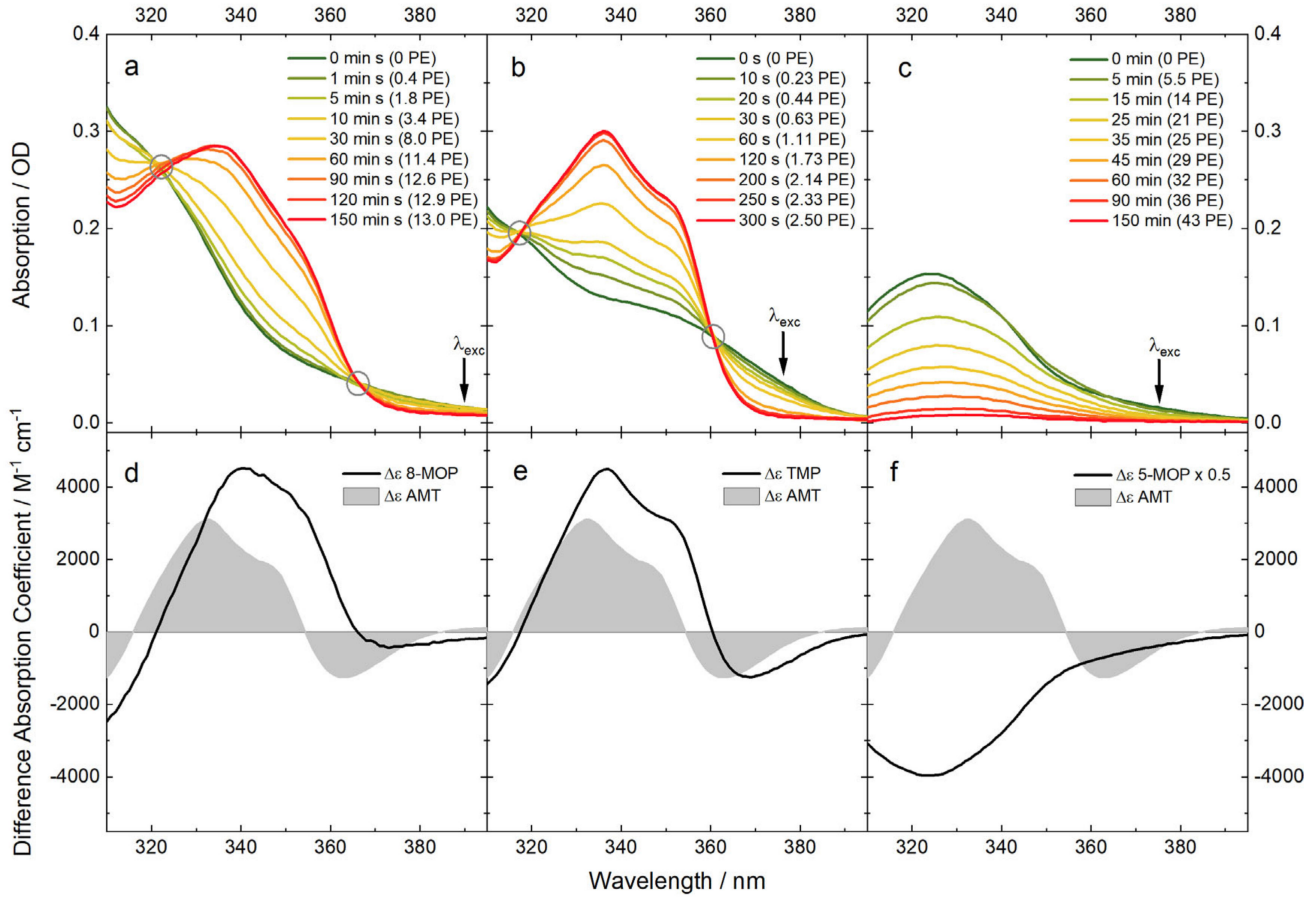
Photoaddition of psoralens to AT-DNA traced by UV/Vis absorption. Irradiation intervals are given in seconds and in photon equivalents (PE). Circles mark isosbestic points. Absorption contributions of the DNA were subtracted: (**a**) Spectra of 8-MOP (33 µM) and AT-DNA (4 mM) in PBS buffer after indicated irradiation times (λ_exc_ = 390 nm, P = 9.5 mW, V = 2.2 mL, d = 1 cm); (**b**) Spectra of TMP (38 µM) and AT-DNA (3.3 mM) in PBS buffer after indicated irradiation times (λ_exc_ = 375 nm, P = 7.5 mW, V = 2.4 mL, d = 1 cm); (**c**) Spectra of 5-MOP (18.4 µM) and AT-DNA (2 mM) in PBS buffer after indicated irradiation times (λ_exc_ = 375 nm, P = 15 mW, V = 2 mL, d = 1 cm); (**d**–**f**) Difference absorption spectra obtained from the data above are compared to the one of AMT with AT-DNA [23].

All three psoralen derivatives absorb light in the UVA range (315–400 nm). The absorption coefficients are high at the lower end of the UVA (5000–15,000 M^−1^ cm^−1^) and low or close to zero around 400 nm. The DNA has very low to zero absorption in this range. The spectrum of 8-MOP intercalated into AT-DNA shows an absorption up to 400 nm (Figure 2a, green). Irradiation at 390 nm causes changes to the absorption spectrum. The irradiation times are converted into photon equivalents (PE) which is a measure of the light dose (see Materials and Methods). A PE value of one implies that each molecule has absorbed one photon. With the irradiation time or PE value, the absorption between 322–366 nm increases and below 322 and above 366 nm decreases. The last spectrum, the spectrum of the photoproduct, features a maximum at 340 nm and a shoulder at ~352 nm. These features indicate the formation of the furan monoadduct [12,33,34,35]. Longer irradiation at 390 nm causes the absorption to decrease throughout the whole UVA spectrum (not shown here). Even though the absorption of the monoadduct is almost zero at 390 nm, the small absorption seems to cause the formation of a secondary photoproduct, presumably a crosslink. Neither the pyrone monoadduct nor the crosslink absorb light in the UVA range [12]. Difference absorption coefficients for the monoaddition were extracted from these spectra. A plot of the absorption versus irradiation time (not shown here) indicates that after ~150 min the monoaddition is terminated. Therefore, the concentration of the photo-product ought to equal the initial concentration of intercalated 8-MOP. From that, the difference absorption coefficients can be computed (Figure 2d). The spectrum has a maximal difference absorption coefficient of ~4000 M^−1^ cm^−1^ and the spectral pattern is red-shifted by 8 nm with respect to that of AMT [23]. With the knowledge of the light power impinging on the sample, a reaction quantum yield $\Phi_{R}$ of 0.04 was computed. The value is higher than yields determined for 8-MOP in calf thymus DNA (0.013 [27], 0.0065 [36] and 0.0046 [37]). The difference could be related to the PET quenching occurring in the DNA-samples bearing guanine. 

Similar spectroscopic signatures are observed for the photoaddition of TMP to AT-DNA (see Figure 2b). Upon irradiation with 375 nm light, absorption increases are observed between 317 and 360 nm and decreases beyond these values. In comparison to 8-MOP, the isosbestic points are better defined. This might be related to a lower propensity of TMP to form crosslinks [33,35]. Assuming that after 300 s or a PE of 2.5 the monoadduct formation has come to a halt, a difference spectrum was computed (see Figure 2e). The spectrum is very similar to the one of the AMT furan monoadduct. A reaction quantum yield $\Phi_{R}$ of 0.4 was determined. Therefore, the psoralene derivative TMP is three times more efficient in binding to AT-DNA then AMT with a quantum yield $\Phi_{R}$ of 0.12 [23]. A higher reaction quantum yield for TMP in comparison to AMT is consistent with the previous studies [27,31]. In these studies, the photoaddition to calf thymus DNA was examined, allowing for no direct comparison of the values.

The spectral changes caused by the irradiation of intercalated 5-MOP are very different from the two discussed above (Figure 2c). The absorption spectrum of intercalated 5-MOP is similar to the one of 8-MOP, although slightly red-shifted. Upon irradiation with 375 nm light a decrease of the absorption in the whole spectral range covered is observed. With increasing irradiation time or PE value the spectrum decays to essentially zero. The difference spectrum is therefore nothing else than the inverted absorption spectrum of intercalated 5-MOP. As such it bears no resemblance with the difference spectra of TMP and 8-MOP. The reaction quantum yield $\Phi_{R}$ was determined to 0.017. The spectral changes may be explained by the formation of the pyrone monoadduct. The pyrone monoadduct does not absorb light in the shown UVA region [38]. In comparison to TMP and 8-MOP there are less studies on the photoproducts of 5-MOP. The results shown here are in agreement with the assumption that for 5-MOP the pyrone side monoadduct seems more favorable [38,39]. 

### 2.3. IR Absorption Signatures of the Photoaddition

**Figure 3:**
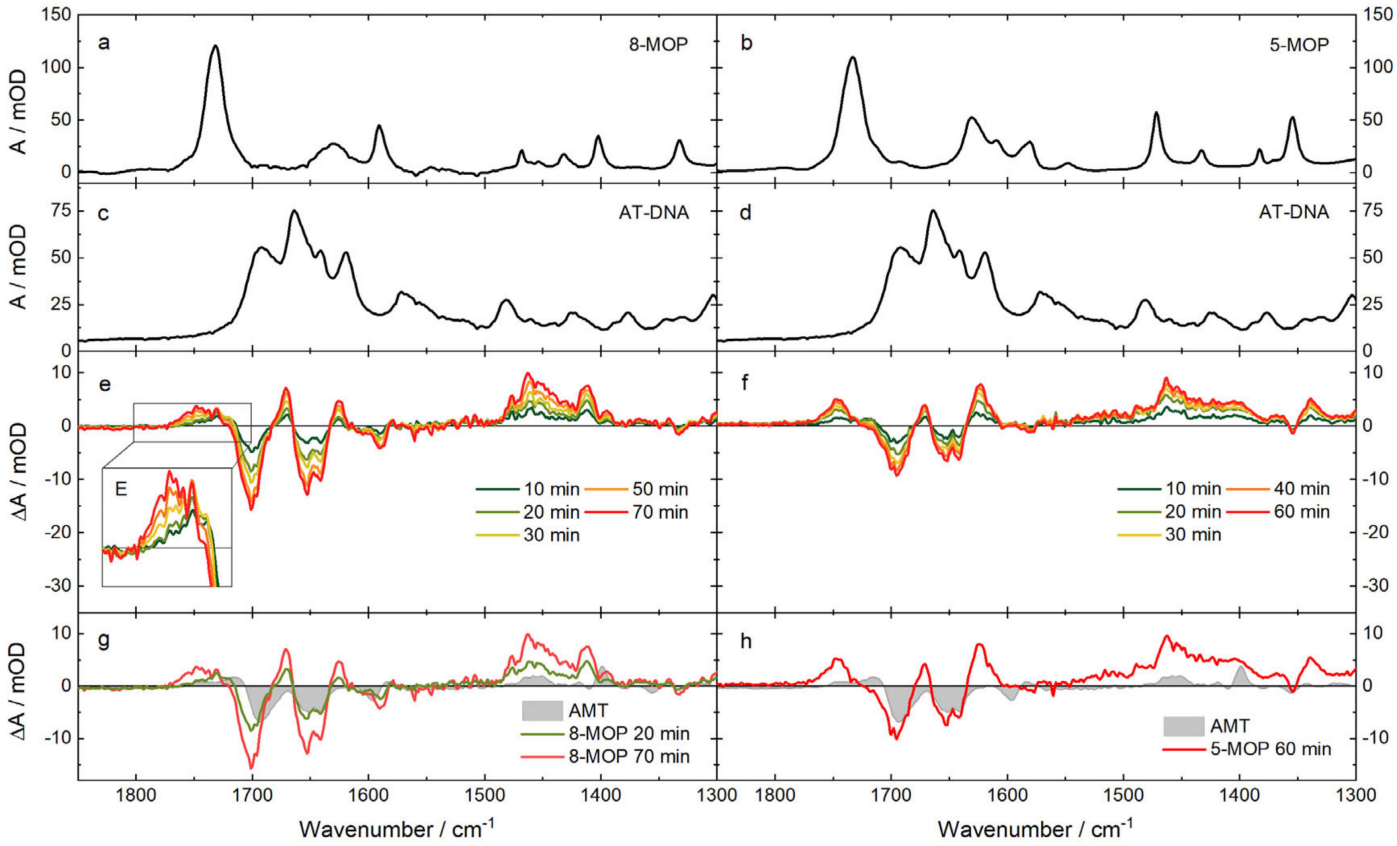
Steady-state IR spectra of the photoaddition of 8-MOP (left) and 5-MOP (right) to AT-DNA: (**a**) 8-MOP (11 mM) in acetonitrile-d3; (**b**) 5-MOP (10 mM) in acetonitrile-d3; (**c**,**d**) AT-DNA (7.7 mM) in PBS buffered D_2_O; (**e**) Difference spectra of the irradiation of 8-MOP (1.3 mM) with AT-DNA (20 mM) in PBS buffered D_2_O (λ_exc_ = 375 nm, P = 26 mW). (**e**) Magnification of the marked range; (**f**) Difference spectra of the irradiation of 5-MOP (0.7 mM) with AT-DNA (20 mM) in PBS buffered D_2_O (λ_exc_ = 375 nm, P = 26 mW); (**g**,**h**) Comparison of difference spectra obtained from the data in (**e**,**f**) with the one of AMT (1.5 mM) with AT-DNA (6.5 mM) (λ_exc_ = 375 nm, P = 15 mW) [23].

Due to the low water solubility, the signals of intercalated TMP in the IR are very small in relation to the noise. Hence, the focus will be on 8-MOP and 5-MOP here. The IR spectra of 8-MOP and 5-MOP without DNA were recorded in deuterated acetonitrile because of solubility reasons (Figure 3a,b). The spectrum of 8-MOP features one very strong vibration band at 1732 cm^−1^. It is attributed to the carbonyl stretching vibration [40,41]. One broad band at 1632 cm^−1^ and a more distinct one at 1591 cm^−1^ are attributed to C=C stretching vibrations. The spectrum of 5-MOP features a strong vibration band at 1733 cm^−1^ which can be attributed to the carbonyl stretching vibration [41]. The bands at 1631, 1609, 1581 and 1549 cm^−1^ can be assigned to C=C stretching vibrations. For the reason of IR transparency AT-DNA and 8-MOP were studied using buffer solutions based on D_2_O. This causes exchangeable protons of DNA to be replaced by deuterons, that is, NH vibrations are not to be expected. AT-DNA features four distinct bands in the upper-frequency range (Figure 3c). The bands at 1693 cm^−1^ and 1663 cm^−1^ can be assigned to carbonyl stretching vibrations of the thymine base and the one at 1641 cm^−1^ and 1619 cm^−1^ to C=C stretching vibrations of the thymine and adenine base respectively [42]. In a solution of 8-MOP (1.3 mM) and AT-DNA (20 mM) in buffer, roughly 95% of 8-MOP is intercalated. When irradiated with an LED emitting at 375 nm, the absorption changes (Figure 3e). Distinct negative absorption changes can be seen at 1701 cm^−1^, 1653 cm^−1^, 1641 cm^−1^ and at 1590 cm^−1^. Sharp positive absorption changes are located at 1671 cm^−1^, 1626 cm^−1^ and 1411 cm^−1^. A broad one is located around 1750 cm^−1^ as well as between 1480–1440 cm^−1^. For the assignment to certain vibrations of the reagents one has to keep in mind, that the IR spectrum of 8-MOP was recorded in deuterated acetonitrile and that the frequencies and transition strengths of the vibrations also differ between intercalated and free psoralen [23]. Negative absorption changes around 1700 cm^−1^ are probably due to the bleach of carbonyl stretching vibrations of 8-MOP as well as thymine. The absence of strong bands of 8-MOP around 1650 cm^−1^ suggests that bleaches at 1653 cm^−1^ and 1641 cm^-1^ are due to thymine. A more detailed assignment can be achieved with the help of quantum chemical calculations (see below). The positive band at around 1750 cm^−1^ features a slight shift to higher wavenumbers with longer irradiation time (Figure 3E). This temporal behavior suggests that this feature is due to secondary photochemistry, that is, crosslink formation. The propensity of 8-MOP for crosslinks was already observed by UV/Vis spectroscopy (see above). The difference spectrum for 8-MOP after 20 min of irradiation is very similar to the one obtained for AMT (Figure 3g), except for positive difference absorption bands at 1671 and 1626 cm^−1^ which are not visible for AMT. After 70 min of irradiation, the spectra differ at 1750 cm^−1^ indicating that crosslink formation is more likely for 8-MOP than for AMT.

In a solution of 5-MOP (0.7 mM) and AT-DNA (20 mM), 99% of 5-MOP is intercalated. At first sight, changes due to irradiation with a 375 nm LED (Figure 3f) are similar to the ones of 8-MOP. A shift in absorption is not visible, which is an indication that no secondary photoreaction took place. The difference spectrum of 5-MOP features similar bleaching bands as the one of AMT (Figure 3h). The positive difference absorption for 5-MOP, which is very similar to the one of 8-MOP after 70 min of irradiation, indicates a different photoreaction, which is presumably the pyrone adduct formation. 

### 2.4. Quantum Chemical Computations of the IR Signatures

Quantum chemical calculations support the interpretation of the experimental infrared spectra. Spectra were computed relying on density functional theory (DFT) using the B3LYP functional and a 6–31 + G* basis set as implemented in Gaussian 09 [43]. The self-consistent reaction field (SCRF) method accounted for the solvent environment implicitly. The DNA environment was not treated explicitly in the computation. Instead, a continuum approach was applied. Hereby, the dielectric constant of pyridine (~13) was chosen to approximate the DNA environment. Water, with a dielectric constant of 78, gave similar results, albeit the carbonyl stretching vibrations being located at slightly lower wavenumbers ($\Delta \tilde{\nu }$~−7 cm^−1^). Acidic protons were exchanged for deuteron. After geometry-optimization, the wavenumbers and IR transitions strength were computed. The harmonic frequencies were scaled by a factor of 0.96 [44]. 

**Figure 4:**
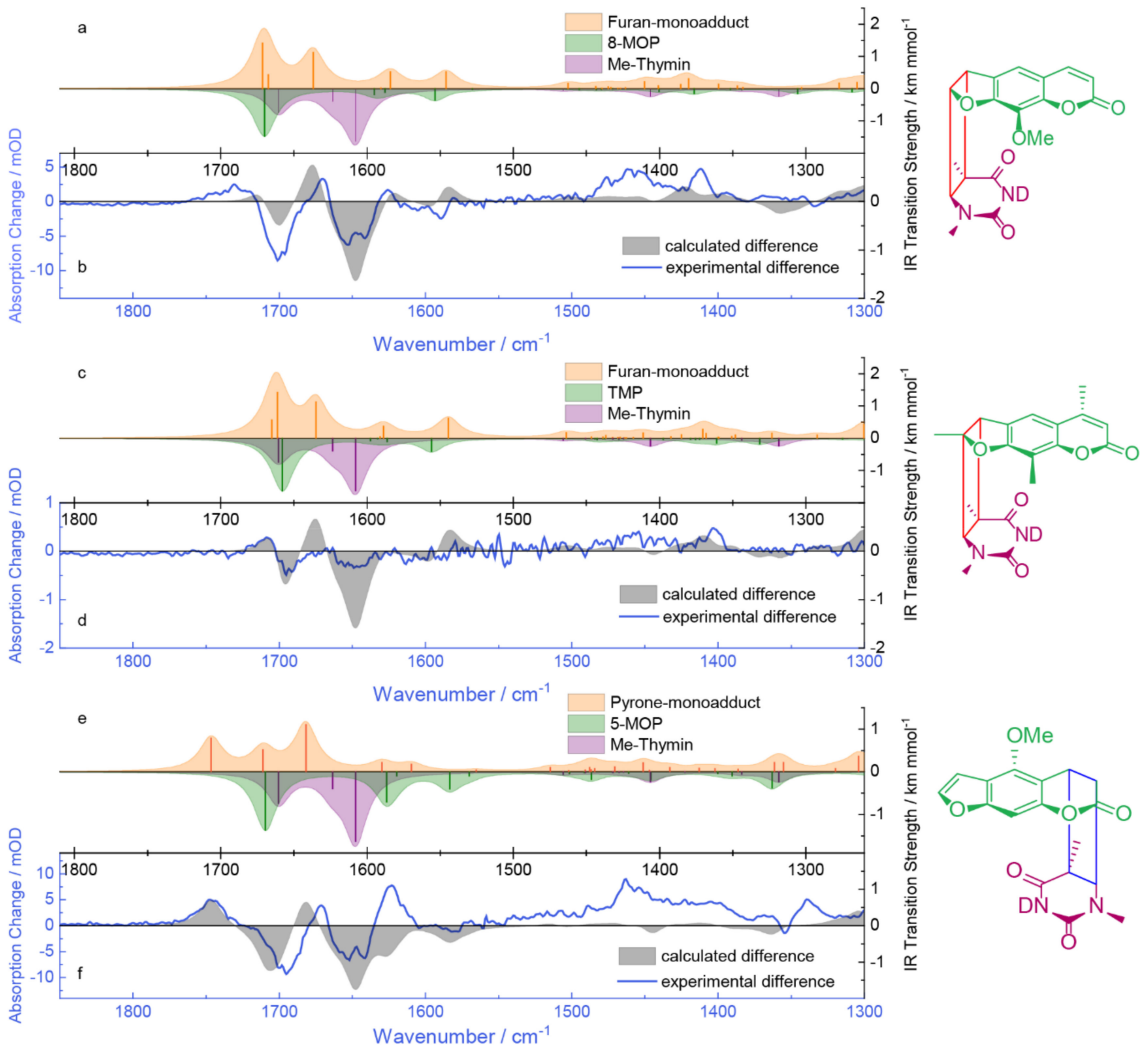
IR signatures of photoaddition computed by quantum chemistry in comparison with the experimental data: (**a**,**c**,**e**) IR spectra of psoralens, 1-methylthymine and their respective photo-products obtained from a DFT calculation with the B3LYP functional and a 6-31 + G* basis set (scaling factor 0.96). Stick spectra were converted into the presented ones by convolution with Lorentzians (full width at half maximum (FWHM) of 20 cm^−1^). The spectra of the psoralens and 1-methylthymine are inverted to highlight possible bleach contributions; (**b**,**d**,**f**) Comparison of calculated difference and experimental difference absorption. The blue X-axes (experimental difference) is shifted by 40 cm^−1^ to higher wavenumbers relative to the black X-axes (computational differences). Structures of the respective photo-products are depicted on the right.

For the calculation of the IR spectra of 8-MOP, Cambridge Structural Database (CSD) entry XANTOX was used as starting geometry [45]. The carbonyl stretching vibration at 1670 cm^−1^ features the highest transitions strength (Figure 4a, green). Three weaker bands are located at 1595, 1588 and 1553 cm^−1^ and can be assigned to C=C stretching vibrations. The computed spectrum shows high similarity to the experimental one in acetonitrile-d3 (compare Figure 3a), though the experimental spectrum is shifted to higher wavenumbers by ~+60 cm^−1^. No attempts to treat (a part of) the double-stranded DNA by quantum chemistry were made. Instead only the IR spectrum of 1-methylthymine was computed. Three bands at 1661, 1624 and 1608 cm^−1^ can be seen. The ones with higher transition strengths are the carbonyl stretching vibrations, while the one at 1624 cm^−1^ can be assigned to a ring deformation mode [42]. Base pairing and stacking as well as other effects influence the vibrations of thymine as part of DNA [46], explaining the difference in frequency and strength of the vibrations in the experimental spectrum of AT-DNA (compare Figure 3b). For the structure of the monoadduct Protein Data Bank (PDB) entry 203D, which is based on nuclear magnetic resonance (NMR) measurements, was used as starting geometry [47]. Hereby, the DNA part was reduced to the respective 1-methylthymine moiety. The carbonyl vibration of 8-MOP at 1671 cm^−1^ experienced almost no shift. The carbonyl vibrations of thymine at 1667 and 1637 cm^−1^ are shifted by +6 and +29 cm^−1^ respectively. At 1591, 1584 and 1546 cm^−1^ the C=C stretching vibrations of 8-MOP can be seen, although the one at 1591 cm^−1^ has lost in strength as it is almost not visible. A synthetic difference spectrum was obtained by subtracting the computed 8-MOP and 1-methylthymine spectrum from the computed adduct spectrum. If one neglects the fact that the computed pattern is located at lower wavenumbers by ~−40 cm^−1^, the two patterns match rather well. The bleach at 1702 cm^−1^, although not as pronounced in the calculation, is due to the shifts in the carbonyl vibration of 8-MOP and one of the carbonyl vibrations of thymine. The positive feature around 1670 cm^−1^ in the experiment can be explained with the shift of the other thymine carbonyl vibration to higher wavenumbers. The bleach contributions at 1653 cm^−1^ and 1641 cm^−1^ seen in the experimental difference spectra are according to the computation due to carbonyl and C=C stretching vibrations of thymine. The fingerprint region is not very well matched, which at this level of calculation is plausible.

In the case of TMP, CSD entry LINTUX served as a starting geometry for calculating IR spectra [48]. The carbonyl stretching vibration is located at 1658 cm^−1^ and features the highest transition strength (Figure 4c, green). Frequencies and transition strengths of C=C vibrations of TMP and TMP as part of the furan monoadduct are very similar to the ones of 8-MOP and its furan monoadduct (compare Figure 3a,c). The synthetic difference spectra are almost identical (compare Figure 3b,d). The experimental difference absorption is, as explained above, due to the low signal to noise ratio not discussed in detail. The region around 1700 cm^−1^ matches rather well, which supports the furan side product formation.

For the calculation of the IR spectra of 5-MOP, CSD entry ARARIW was used as starting geometry [49]. The carbonyl stretching vibration at 1669 cm^−1^ features the highest transition strength (Figure 4e, green). Bands at 1587, 1580, 1543 and 1530 cm^−1^ are attributed to C=C vibrations. All these bands can be found in the experimental spectrum in acetonitrile-d3 as well (compare Figure 3b). The starting geometry for the pyrone monoadduct was extracted from PDB entry 204D [47]. The carbonyl vibration of 5-MOP experiences a major shift (+37 cm^−1^). The thymine vibrations are shifted by +10 and +33 cm^−1^ respectively. Bands at 1590 and 1570 cm^−1^ are attributed to C=C stretching vibrations of 5-MOP. The synthetic difference spectrum shows high similarity to the experimental one, with exception of the fingerprint region. The positive difference absorption around 1750 cm^−1^ is due to the shift of the 5-MOP carbonyl vibration, which cannot be seen in the calculation of the furan monoadducts. Hence, it can be seen as an indicator of the photoaddition on the pyrone side. 

### 2.5. Nanosecond Transient UV/Vis Absorption Signatures of the Photoaddition

Solutions of 8-MOP and TMP with AT-DNA were excited with UVA laser pulses and probed in the UV/Vis region. Due to the lack of spectroscopic signatures of the 5-MOP photo-product in the accessible UV/Vis region, only 8-MOP and TMP are treated in the following. 

**Figure 5:**
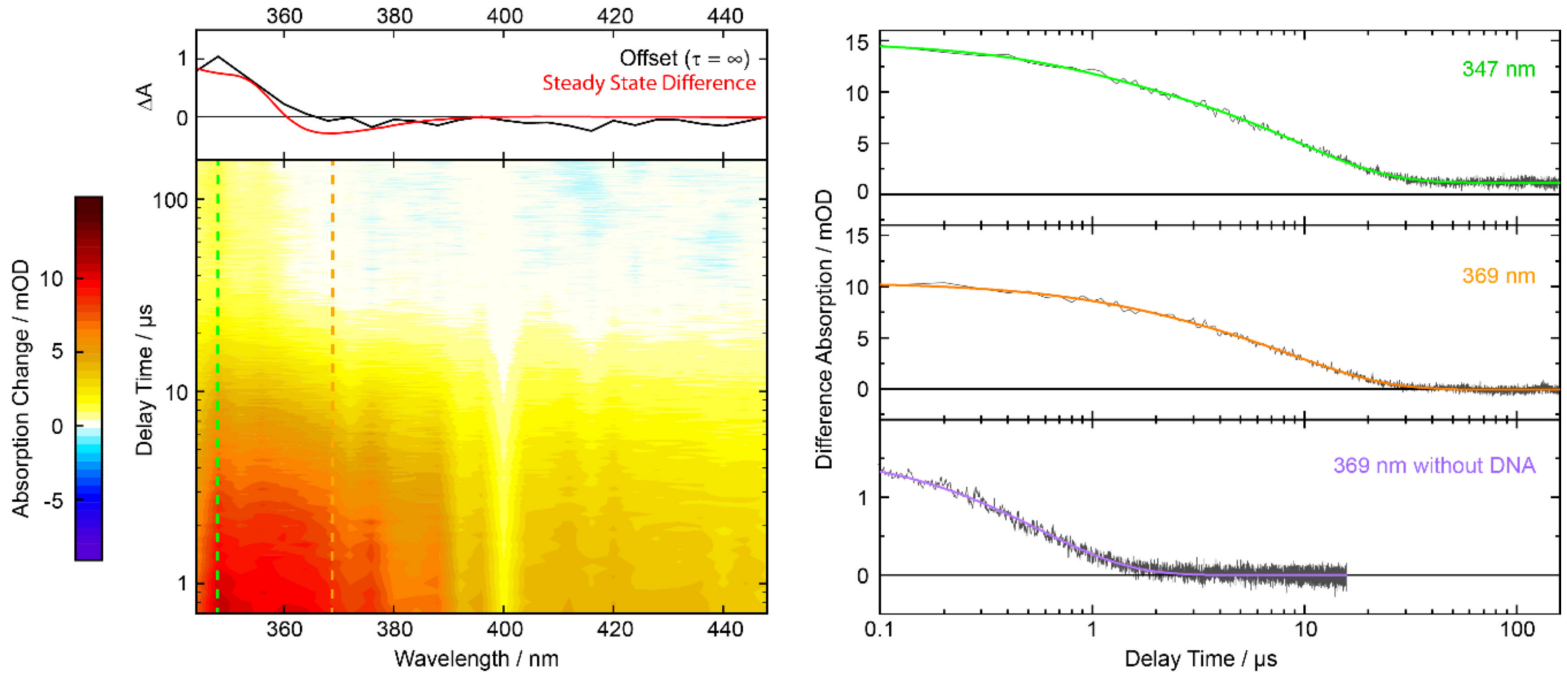
Nanosecond transient absorption of 8-MOP (0.3 mM) with AT-DNA (3 mM) in aerated buffer solution after excitation at 355 nm. Left: The transient absorption in the contour plot is color-coded. The dashed lines mark the respective time trace shown on the right. On top is the offset spectrum in comparison with the steady-state signature (red, scaled to match the offset spectrum) after irradiation at 390 nm. Right: Time traces at 347 and 369 nm. The third time trace shows 8-MOP (0.2 mM) without DNA in water. The colored lines show bi-exponential (green and orange) or single exponential (violet) fits of the data.

A solution of 8-MOP and AT-DNA in buffered water was excited with nanosecond laser pulses centered at 355 nm (Figure 5). At these concentrations ~70% of 8-MOP is intercalated. For the absorptions employed, the detection wavelengths below 340 nm were not accessible. The spectral pattern around time zero, featuring an absorption band peaking at ~360 nm, is similar to the one reported for the triplet state of non-intercalated 8-MOP [50,51]. So, it is very likely that the time zero signature is due to the triplet state of intercalated and partly due to non-intercalated 8-MOP. For the conditions employed here, the measurement reveals a triplet decay time of 0.6 µs for non-intercalated 8-MOP (see Figure 5, violet). The value is in good agreement with the literature [51], if one takes the intrinsic first-order decay (2.5 × 10^5^ s^-1^), self-quenching (3.8 × 10^9^ M^−1^ s^−1^) and quenching by oxygen (4 × 10^9^ M^−1^ s^−1^) into account. For non-intercalated 8-MOP the signal at large delay times is essentially zero. For these delay times, the intercalated 8-MOP features a distinct difference absorption signal at wavelengths smaller than 360 nm.

The decay pattern of intercalated 8-MOP (see Figure 5, green and orange) is also in stark contrast to the behavior of non-intercalated one (Figure 5, violet). The decay is bi-exponential with time constants of $\tau_{1}$ = 1 µs and $\tau_{2}$ = 10 µs (values obtained by global analysis). In the time traces for intercalated 8-MOP, no indications for a tri-exponential decay are observed. One could therefore reason, that the time constant $\tau_{1}$ of 1 µs is due to residual non-intercalated 8-MOP which features a time constant close to 1 µs. However, in an oxygen-saturated solution (~1 bar) of 8-MOP and AT-DNA (data not shown), the 1 µs time constant persists. Since one expects significant oxygen quenching for non-intercalated 8-MOP [52], the time constant $\tau_{1}$ of ~1 µs can be attributed to intercalated 8-MOP. 

**Figure 6:**
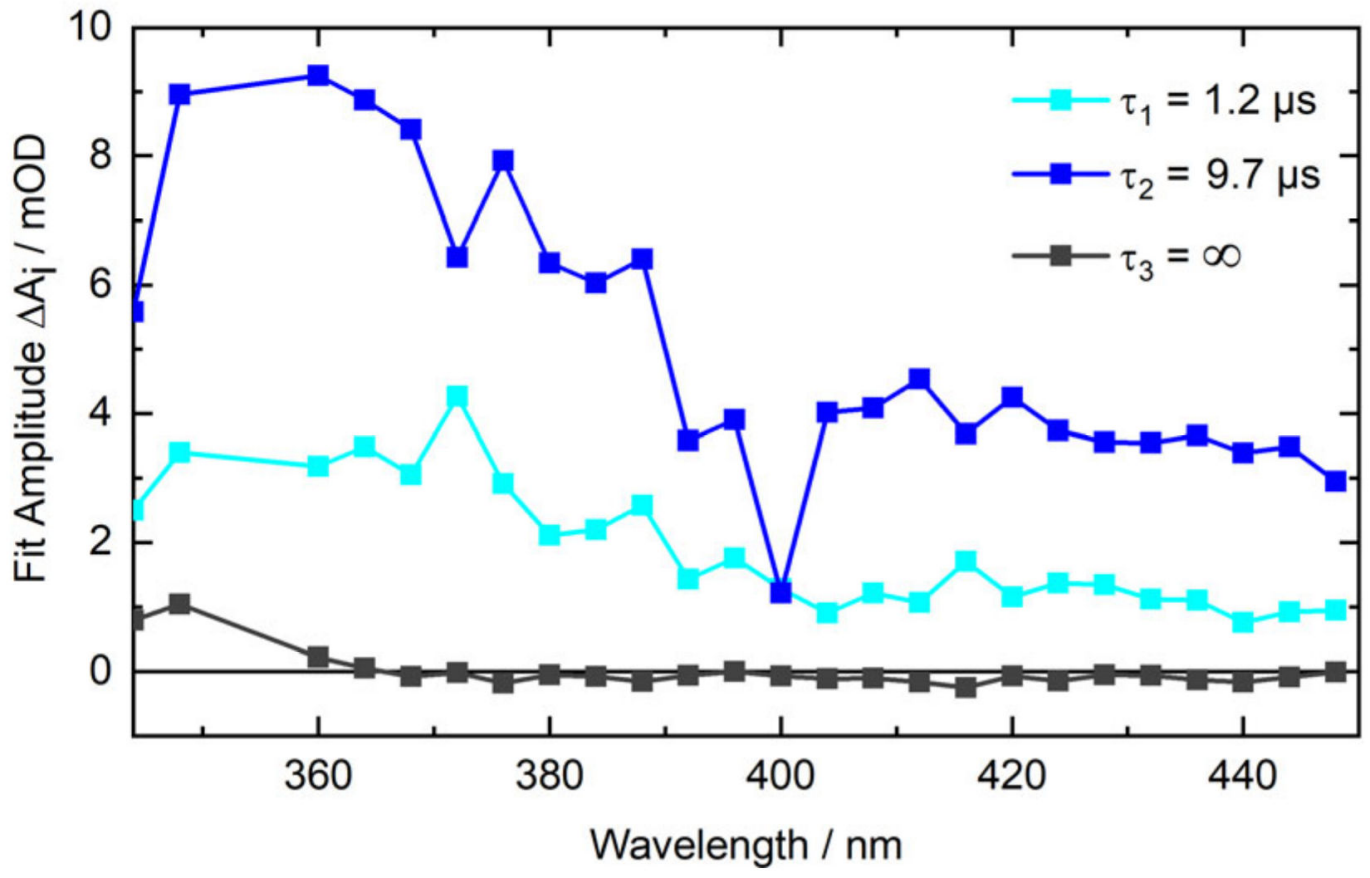
DADS from a multi-exponential global analysis of the nanosecond transient absorption data of 8-MOP with AT-DNA. The time trace at excitation wavelength 355 nm was not included in the fit.

The respective decay associated difference spectra (DADS) of the data above are shown in Figure 6. Both bear resemblance with the 8-MOP triplet spectrum [50,51]. The global analysis also yields an offset spectrum ($\tau_{3}=\infty$) which matches the steady-state difference spectrum of the photo-addition (cf. Figure 5, top).

**Figure 7:**
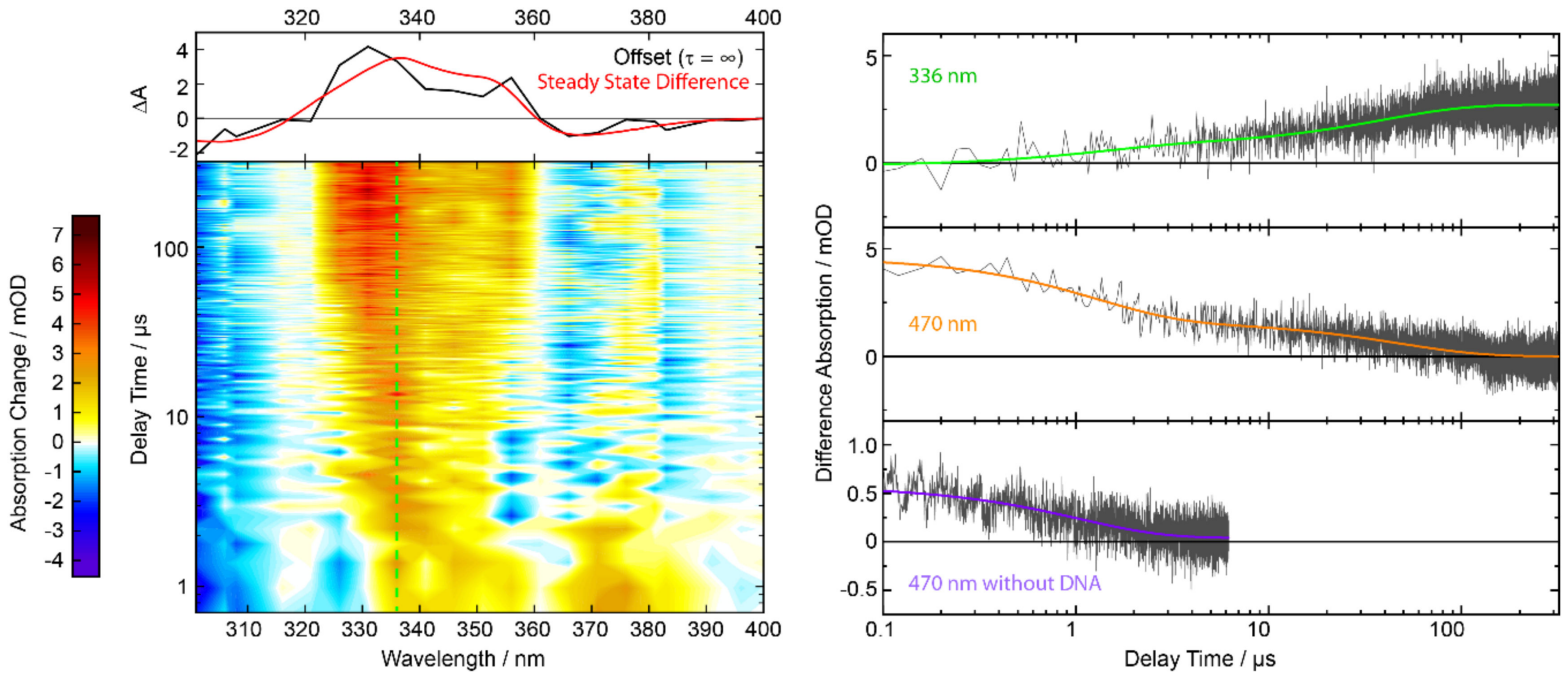
Nanosecond transient absorption of TMP (17 µM) with AT-DNA (1.6 mM) in aerated buffer solution after excitation at 355 nm. Left: The transient absorption in the contour plot is color-coded. The green line marks the respective time trace (336 nm) shown on the right. On top is the offset spectrum in comparison with the steady-state signature (red, scaled to match the offset spectrum) after irradiation at 375 nm. Right: Time traces at 336 and 470 nm. The third time trace shows TMP (6 µM) without DNA in water. The colored lines show bi-exponential (green and orange) or single exponential (violet) fits of the data.

The photoreaction of TMP and AT-DNA was traced by the same approach. A solution of TMP and AT-DNA in buffered water was excited with nanosecond laser pulses centered at 355 nm (Figure 7). Due to the low solubility of TMP, signal levels are smaller than those of 8-MOP. Furthermore, the higher reaction quantum yield $\Phi_{R}$ of TMP compared to 8-MOP implies that the reactants are converted to photo-product after fewer scans. We, therefore, covered only the spectral region centered around 350 nm. In this range, the signature of the photo-product is expected. Around time zero a negative transient absorption for wavelengths smaller than ~320 nm is observed. This is due to ground state bleach. For longer wavelengths, a positive signal is detected. The signature is in line with the triplet signatures of non-intercalated TMP. The respective spectra feature a maximum around 470 nm [17]. Indeed, a single wavelength scan at 470 nm (see Figure 7, orange) reveals a relatively strong time zero signal. For non-intercalated TMP the signal decays to essentially zero within ~1 µs. The decay for intercalated TMP proceeds bi-exponentially with time constants of $\tau_{1}$~1 µs and $\tau_{2}$~40 µs. This decay goes along with the built-up of an offset signal between 320–360 nm, which matches the steady-state difference spectrum of the photo-addition. Relative to the time zero signal the offset signal is higher than the one of 8-MOP. This matches the expectation based on the reaction quantum yield $\Phi_{R}$. 

## 3. Discussion

Our previous results on the furan side photo-addition of AMT to AT-DNA [23] showed that this addition proceeds via the local triplet state of AMT and a triplet biradical. The present study indicates that this mechanism also applies to 8-MOP and TMP.

For 8-MOP intercalated into AT-DNA a bi-exponential decay with time constants of $\tau_{1}$~1 µs and $\tau_{2}$~10 µs were observed. We assign the time constant $\tau_{1}$ to the decay of the 8-MOP triplet state, which presumably goes along with the formation of a triplet biradical in which the psoralen at 5′ position (see Scheme 1) is connected with the thymine moiety at position 6 by a single bond. The time constant $\tau_{2}$ would then be associated with the decay of the biradical and formation of the final product. Seemingly in conflict with this interpretation is the observation that the spectral signatures do not change much during the $\tau_{1}$ process (cf. Figure 6). Presumably, this is due to similar spectral signatures of the local triplet state and the triplet biradical. Such similarity was already observed for AMT and AT-DNA [23]. For this system, the intermediary of a triplet biradical found strong support from time-resolved IR spectroscopy. 

For a triplet state as a precursor, the reaction quantum yield $\Phi_{R}$ is given by the triplet quantum yield $\Phi_{T}$ times the reaction efficiency of the triplet state $\eta_{R}^{T}\leq 1$, that is, $\Phi_{R}=\Phi_{T}\cdot \eta_{R}^{T}$. The reaction quantum yield $\Phi_{R}$ for the addition of 8-MOP to AT-DNA was determined to be 0.04. The triplet yield $\Phi_{T}$ of non-intercalated 8-MOP was reported to be 0.06 [51]. If this value also applies to intercalated 8-MOP, the efficiency $\eta_{R}^{T}$ amounts to 0.67. A similar value is derived from a different approach. The efficiency $\eta_{R}^{T}$ can be obtained from

(2) $$\eta_{R}^{T}=\frac{c_{PP}}{c_{T}}=\;\frac{c^{t=\infty }}{c^{t=0}},$$

$c_{PP}$ is the concentration of the photo-product in the time-resolved experiment. This concentration is measured at “infinite” times ($c^{t=\infty }$). $c_{T}$ is the triplet concentration which is measured at time zero ($c^{t=0}$). Concentrations $c_{PP}$ and $c_{T}$ can be obtained from the respective difference absorption signals ΔA and coefficients Δε,

(3) $$\eta_{R}^{T}=\frac{\Delta A_{347\;\mathrm{nm}}^{t=\infty }}{\Delta \varepsilon_{347\;\mathrm{nm}}^{PP}}\cdot \frac{\Delta \varepsilon_{370\;\mathrm{nm}}^{T}}{\Delta A_{370\;\mathrm{nm}}^{t=0}}.$$

For the photo-product the signal $\Delta A_{347\;\mathrm{nm}}^{t=\infty }$ and coefficient $\Delta \varepsilon_{347\;\mathrm{nm}}^{PP}$ at 347 nm, as determined here, were inserted. For the triplet state the signal $\Delta A_{370\;\mathrm{nm}}^{t=0}$, corrected for the intercalated fraction and coefficient $\Delta \varepsilon_{370\;\mathrm{nm}}^{T}$ at 370 nm [51] were used. The coefficient $\Delta \varepsilon_{370\;\mathrm{nm}}^{T}$ refers to 8-MOP in water. With these values an efficiency $\eta_{R}^{T}$ of ~0.6 results. This implies that–compared to AMT–the 8-MOP triplet is somewhat more reactive ($\eta_{R}^{T}$ of AMT: 0.3–0.4 [23]). Due to the smaller triplet yield $\Phi_{T}$ of 8-MOP its overall reaction quantum yield $\Phi_{R}$ is smaller ($\Phi_{R}$ of AMT: 0.12). 

Also, for TMP a bi-exponential decay pattern is observed. The time constants of $\tau_{1}$ = 1 µs and $\tau_{2}$ = 40 µs are very close to the one reported for AMT intercalated into AT-DNA [23]. Taking the structural similarity of AMT and TMP into account, this is not surprising. It is therefore also likely that like with intercalated AMT the time constant $\tau_{1}$ describes the decay of the local triplet state accompanied by the biradical formation. The time constant $\tau_{2}$ is therefore assigned to the biradical decay and the formation of the photo-product. The high reaction quantum yield $\Phi_{R}$ of 0.4 is somewhat surprising. The reported triplet yield $\Phi_{T}$ of non-intercalated TMP is ~0.1 in methanol [17]. This would imply an unphysical efficiency $\eta_{R}^{T}>1$. Our present interpretation of this is that the triplet yield $\Phi_{T}$ of intercalated TMP is substantially larger than the one for non-intercalated TMP. Indications for that were already observed for AMT [23].

The behavior of 5-MOP is qualitatively different for the one of AMT, 8-MOP and TMP. In line with earlier studies, our UV/Vis and IR measurements indicate that the photo-addition proceeds via the pyrone side of the psoralen and not the furan side. Unfortunately, due to the lack of a spectroscopic signature of this adduct in the accessible UV/Vis range, the formation could not be traced in real-time. Time-resolved IR experiments ought to be conducted to clarify this in the future. 

## 4. Materials and Methods 

### 4.1. Samples

8-MOP and 5-MOP were purchased from TCI (Tokyo, Japan, >98%) and TMP from Sigma-Aldrich (Steinheim, Germany, ≥98%). The lyophilized oligonucleotide 5′-(TA)_20_-3′ was purchased from Sigma-Aldrich. The manufacturer purified the sample by HPLC. Annealing of the oligonucleotide strands in solution was performed within 24 h before the measurements by heating the solution in a water bath up to 93 °C and letting it cool down to room temperature within several hours. Solvents used were pure water (Fisher Chemical, Loughborough, UK, HPLC gradient grade), deuterium oxide (Deutero GmbH, Kastellaun, Germany, 99.9% D) and acetonitrile-d3 (Sigma-Aldrich, ≥99.8% D). Solutions of the oligonucleotides were buffered with PBS (Sigma Aldrich, one tablet dissolved in 200 mL yielded 10 mM phosphate buffer, 2.7 mM potassium chloride, 137 mM sodium chloride, pH 7.4 at 25 °C). 

### 4.2. Steady-State Spectroscopy

Absorption spectra in the UV/Vis were recorded with a Lambda 19 spectrometer from Perkin Elmer. Fused silica cuvettes with path lengths of 0.1, 1 and 5 cm from Hellma were employed. Absorption spectra in the IR were recorded with an FT-IR-spectrometer Vertex 80v from Bruker Optik. A custom-made cuvette with a Teflon spacer for a path length of 0.1 mm was used. CaF_2_ windows of 3 mm thickness from Korth Kristalle were employed. The spectra were corrected for the absorption of aqueous vapor and HDO, if necessary. All measurements were performed at room temperature. 

For the irradiation in the steady-state experiments, LEDs emitting at 375 nm or 390 nm were used. For the determination of the quantum yield in the UV/Vis experiments the solutions were stirred while irradiating. With the light power P and the irradiation time t photon equivalents *PE* were computed via the equation:

(4) $$PE\left(t\right)=\frac{n_{abs}\left(t\right)}{n_{Pso}}=\frac{I_{0}\cdot \int_{0}^{t}\left(1-10^{-A_{Ex}\left(t\right)}\right)\;dt}{n_{Pso}}=\frac{P\cdot \int_{0}^{t}\left(1-10^{-A_{Ex}\left(t\right)}\right)\;dt}{h\cdot \frac{c}{\lambda_{Ex}}\cdot N_{A}\cdot n_{Pso}}.$$

Here, $n_{abs}$ is the amount of the absorbed photons and $n_{Pso}$ the amount of psoralen molecules. $A_{Ex}$ refers to the absorption at excitation wavelength $\lambda_{Ex}$. h is defined as the Planck’s constant, c as the speed of light and $N_{A}$ as Avogadro’s number. A PE of one implies that on average each psoralen molecule has absorbed one photon. 

### 4.3. Nanosecond Transient Absorption in the UV/Vis

The nanosecond transient absorption data were acquired with a laser flash photolysis spectrometer LP980 from Edinburgh Instruments in a right-angle geometry. The frequency tripled output (355 nm) of a Nd:YAG laser (Spitlight 600, InnoLas, Germany) with a repetition rate of 5 Hz and a pulse duration of 12 ns (FWHM) was utilized for photoexcitation. The excitation energy ranged from 4–17 mJ per pulse. The diameter of the pump beam was ~8 mm. A pulsed xenon lamp (Osram XBO 150 W/CR OFR) generated the probe light. Fused silica flow-through cuvettes from Hellma with different path lengths in pump and probe direction were employed. To gain the best signal to noise ratio while keeping the turnover rate low, cuvettes with different dimensions (1.5 × 3 mm, 2 × 10 mm or 5 × 10 mm) were used, depending on the sample. The transmitted probe light was dispersed by a grating monochromator and detected by a photomultiplier (Hamamatsu, Japan, R928). The signal was digitized by an oscilloscope (MDO 3022, Tektronix, Beaverton, OR, USA) and the absorption change was calculated based on measurements with and without laser excitation. For every time trace three sets of 8 consecutive measurements were averaged. If indicated, solutions were purged with oxygen or nitrogen (99.999%, Air Liquide, Düsseldorf, Germany). The measurements were performed at 17 °C. 

### 4.4. Data Analysis

The time-resolved data were analyzed with a global multi-exponential fit function

(5) $$\Delta A\left(\lambda ,t\right)=IRF⊗\overset{n}{\underset{i=1}{\sum }}\Delta A_{i}\left(\lambda \right)\cdot e^{-\frac{t}{\tau_{i}}},$$

which is convoluted with an instrumental response function (IRF). The IRF was approximated by a Gaussian with an FWHM of 0.1 µs. The fit yields time constants $\tau_{i}$ and decay associated difference spectra (DADS) $\Delta A_{i}\left(\lambda \right)$ [53].

## 5. Conclusions

The photoaddition of three pharmaceutical psoralens (5-MOP, 8-MOP and TMP) to AT-DNA was studied by steady-state and time-resolved spectroscopy. IR spectroscopy proved to be useful in distinguishing pyrone side (5-MOP) and furan side (8-MOP and TMP) photoadditions. The furan side additions were shown to proceed via a local triplet state and a triplet biradical. The present results, thus, underscore the importance of triplet states for the photo-reactivity. Once this state is populated, the investigated psoralens add to thymine with high efficiency. So, when optimizing psoralens for the PUVA therapy, a small dissociation constant K_D_ [54], a low propensity for PET quenching by guanine [25] and a high triplet yield should be aimed at. Optimizations along these lines are presently undertaken by us.
